# Tailoring Antiplatelet Therapy Duration After PFO Closure: Insights From the PROLONG Registry

**DOI:** 10.1016/j.jacadv.2026.102943

**Published:** 2026-06-30

**Authors:** Carlo Gaspardone, Daniela Trabattoni, Daniele O. d’Atri, Chiara Fraccaro, Alessandro Beneduce, Michele Morosato, Paolo Costa, Giulio Falasconi, Riccardo Mazza, Riccardo Mager, Andrea Donti, Francesco Saia, Evelina Toscano, Filippo Scalise, Giuseppe Patti, Roberto Nerla, Fausto Castriota, Carmine Musto, Maurizio Paciaroni, Francesco Burzotta, Gianluca Ricchetti, Ciro S. Vella, Luca A. Ferri, Giorgio Bassanelli, Marco B. Ancona, Filippo Russo, Barbara Bellini, Giuseppe Barone, Massimo Slavich, Francesca Napoli, Gabriele Paci, Francesco Versaci, Carlo Pappone, Massimo Mancone, Giuseppe Tarantini, Alaide Chieffo, Marco Metra, Achille Gaspardone, Matteo Montorfano, Cosmo Godino

**Affiliations:** a Interventional Cardiology Unit, IRCCS San Raffaele Scientific Institute, Milan, Italy; b Department of General Surgery and Surgical Specialty Paride Stefanini, Sapienza University of Rome; c IRCCS Centro Cardiologico Monzino, Milan, Italy; d Vita-Salute San Raffaele University, IRCCS San Raffaele Scientific Institute, Milan, Italy; e Department of Cardiac, Thoracic, Vascular Science and Public Health, University of Padova, Italy; f Cardiac Surgery Unit, IRCCS San Raffaele Scientific Institute, Milan, Italy; g Institute of Cardiology, ASST Spedali Civili, University of Brescia, Brescia, Italy; h ⁠Facultat de Medicina i Ciències de la Salut, Universitat de Barcelona (UB), Barcelona, Spain; i IRCCS University Hospital of Bologna, Policlinico S. Orsola, Bologna, Italy; j Department of Interventional Cardiology, Policlinico di Monza, Monza, Italy; k Division of Cardiology, Maggiore della Carità Hospital, Novara, Italy; l Cardiology Unit, Maria Cecilia Hospital GVM Care and Research, Cotignola, Italy; m Division of Cardiology, San Camillo Hospital, Rome, Italy; n Department of Neurosciences and Rehabilitation, University of Ferrara, Ferrara, Italy; o Department of Cardiovascular Sciences, IRCCS A. Gemelli University Polyclinic, Rome, Italy; p Division of Cardiology, Santa Maria Goretti Hospital, Latina, Italy; q Department of Arrhythmology, IRCCS San Donato, Milan, Italy; r Umberto I Hospital, Sapienza University of Rome, Rome, Italy; s Division of Cardiology, S. Eugenio Hospital, Rome, Italy

**Keywords:** cryptogenic stroke, dual antiplatelet therapy, patent foramen ovale, residual shunt, RoPE score, transcatheter occluder device

## Abstract

**Background:**

The optimal duration of antiplatelet therapy (APT) after patent foramen ovale (PFO) device closure remains uncertain.

**Objectives:**

This study aimed to evaluate the impact of APT duration on long-term outcomes after PFO closure.

**Methods:**

PROLONG (PFO Transcatheter Occlusion Long-Term Outcomes National Group; NCT06504121) is a multicenter retrospective registry of patients who underwent PFO device closure between 1999 and 2013 at 12 Italian centers. This analysis included patients with successful PFO closure, no significant residual shunt, and no other indication for long-term antithrombotic therapy. Patients were categorized by APT duration after PFO closure in the discontinuation group (≤12 months) or the continuation group (>12 months). The primary outcome was net adverse clinical events (NACE), a composite of ischemic events (ischemic stroke, transient ischemic attack, or systemic embolism) and major bleeding (Bleeding Academic Research Consortium ≥3). Inverse probability of treatment weighting was applied for baseline confounders.

**Results:**

Among 940 patients (mean age 47 ± 12 years; 55% women) followed for 14.0 ± 3.1 years, the cumulative incidence of NACE was 3.6% in the APT discontinuation group and 7.2% in the APT continuation group (adjusted HR [aHR]: 0.71; 95% CI: 0.38-1.37; *P* = 0.31). Ischemic events were similar (3.1% vs 4.3%; *P* = 0.66), while major bleeding was lower in the APT discontinuation group (0.9% vs 2.8%; *P* = 0.014). APT discontinuation was associated with lower NACE in patients with Risk of Paradoxical Embolism (RoPE) score ≥7 (aHR: 0.32; 95% CI: 0.11-0.90; *P* = 0.039), but not in those with RoPE <7 (aHR: 1.07; 95% CI: 0.49-2.35; *P* = 0.89; *P* for interaction = 0.089).

**Conclusions:**

In patients with a RoPE score ≥7, early discontinuation of APT after effective PFO closure was associated with a lower incidence of NACE at long-term follow-up.

Patent foramen ovale (PFO) device closure is the standard treatment for patients with cryptogenic embolism and PFO.^1^, ^2^, ^3^ Following device implantation, dual antiplatelet therapy (DAPT) is routinely prescribed to prevent device-related thrombosis, which may occur before complete endothelialization of the occluder, a process typically completed within 3 to 6 months.^4^, ^5^, ^6^, ^7^ Accordingly, current guidelines consistently recommend DAPT for up to 6 months after PFO closure. After this initial period, single antiplatelet therapy (SAPT) is commonly continued, but its optimal duration remains uncertain due to limited evidence and lack of consensus.^1^, ^2^, ^3^^,^^8^^,^^9^ Prolonged SAPT has been advocated to prevent late device-related thrombosis, a rare but recognized complication, and recurrent ischemic events in patients where the causal role of the PFO remains uncertain.^7^^,^^10^, ^11^, ^12^, ^13^ However, extended SAPT is not without risk, as its long-term continuation is associated with increased bleeding complications.^10^^,^^12^^,^^13^ The Risk of Paradoxical Embolism (RoPE) score, a validated tool estimating the probability that a PFO is causally related to the index embolic event, may help guide tailored APT duration decisions.^14^^,^^15^

Against this background, this study evaluated the impact of APT duration on long-term outcomes after PFO device closure, leveraging data from the multicenter PROLONG (PFO Transcatheter Occlusion Long-Term Outcomes National Group) registry.^16^

## Methods

The PROLONG registry is a multicenter retrospective registry designed to evaluate long-term outcomes after PFO device closure. It includes 1,245 patients who underwent PFO closure for cryptogenic embolism across 12 Italian hospitals between 1999 and 2013, with a mean follow-up of 14.5 ± 2.4 years. Comprehensive details of the PROLONG registry have been previously published, and the study is registered at ClinicalTrials.gov (NCT06504121).^16^

**Figure 1:**
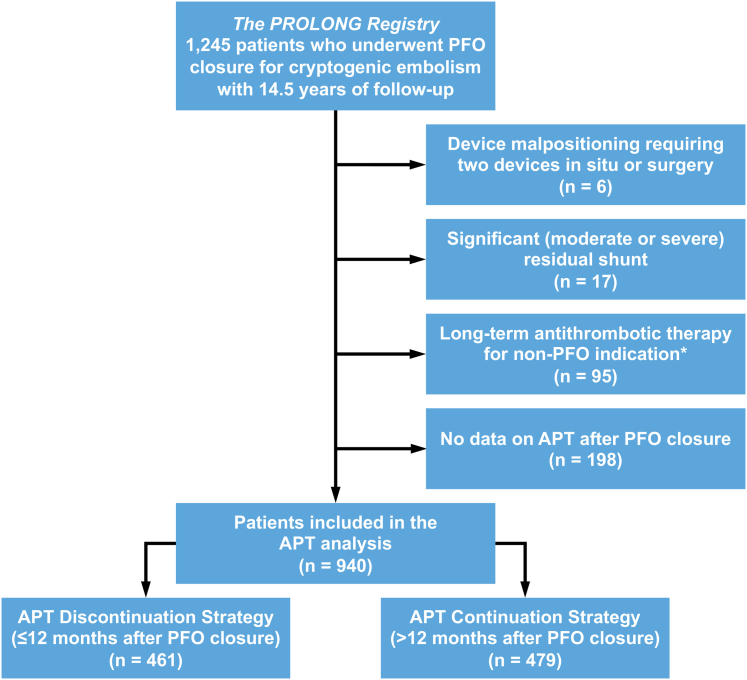
**Study Flowchart** ∗Patients with a newly emerging indication for long-term antithrombotic therapy during follow-up were censored at the time of diagnosis. Note: 11 patients met multiple exclusion criteria APT = antiplatelet therapy; PFO = patent foramen ovale; PROLONG = PFO Transcatheter Occlusion Long-Term Outcomes National Group.

From the PROLONG registry, only patients with successful implantation of a single device, no significant (moderate or severe) residual shunt, no other indication for long-term antithrombotic therapy (either anticoagulant or antiplatelet), and complete data on postprocedural antithrombotic therapy were included (Figure 1). Additionally, to minimize confounding, patients who developed a new indication for long-term antithrombotic therapy during follow-up were censored at the time of diagnosis. Patients were categorized according to APT duration following device implantation as managed with an APT discontinuation strategy (all APT discontinued within 12 months, with patients being free from any antiplatelet drug thereafter) or an APT continuation strategy (SAPT continued beyond 12 months). In both groups, DAPT was typically administered during the early postimplantation period, followed by SAPT. Duration and type of APT, as well as all other management decisions in this retrospective multicenter registry, were at the discretion of the treating physician and participating center.

The primary outcome was the composite of net adverse clinical events (NACE), defined as the occurrence of recurrent ischemic events (ischemic stroke, transient ischemic attack [TIA], or systemic embolism [SE]) or major bleeding. All neurologic and bleeding events occurring during follow-up were classified according to the TOAST (Trial of Org 10172 in Acute Stroke Treatment) and Bleeding Academic Research Consortium (BARC) classifications, respectively.^17^^,^^18^ Major bleeding events were defined as BARC type ≥3. As previously detailed, cryptogenic embolism was defined as ischemic stroke, TIA, SE, or silent ischemic lesions detected by magnetic resonance imaging.^16^

The study was conducted in accordance with the Declaration of Helsinki. The study was approved by the local ethics committees. Informed consent was waived due to the retrospective nature of the study. All data were anonymized prior to analysis.

### Statistical analysis

**Figure 2:**
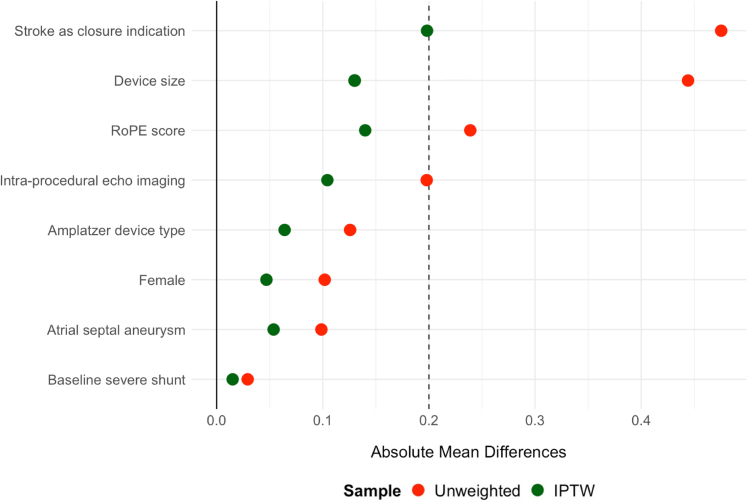
**Covariate Balance Before and After Inverse Probability of Treatment Weighting** Red dots indicate standardized mean differences at baseline; green dots indicate standardized mean differences after weighting. The dashed line indicates the threshold of 0.2; values below this threshold are indicative of an appreciable reduction in imbalance between groups. IPTW = inverse probability of treatment weighting; RoPE = risk of paradoxical embolism.

Categorical variables are reported as counts and percentages, and continuous variables as mean ± SD or median with 25th-75th percentiles (Q1-Q3), as appropriate. Group comparisons were performed using the Student’s *t*-test or Wilcoxon rank sum test for continuous variables, and the chi-square test for categorical variables. Events are reported as absolute numbers, rates (per 100 patient-years), and cumulative incidence function, accounting for death as a competing risk. Time-to-event analyses are presented using both the Kaplan-Meier method (compared with log-rank tests) and cumulative incidence function estimates (compared with Gray’s test). Given the observational nature of the study, potential confounding factors could bias the relationship between APT duration and outcomes; therefore, a propensity score was developed. Variables for the propensity score model were selected based on their clinical relevance or their known or plausible associations with the outcomes, according to literature data (Table 1). Rather than including age and individual cardiovascular risk factors separately, the RoPE score was used as a single continuous variable that incorporates these components, thereby ensuring model stability and avoiding overfitting. Missing propensity covariate data were handled using multiple imputation by chained equations, generating 20 imputed data sets, using the R package mice. A propensity score was derived using logistic regression, with the selected covariates as independent variables and the treatment group as the binary outcome. Applying inverse probability of treatment weighting (IPTW), each patient was assigned a weight derived from the propensity score using the R package WeightIt. To mitigate the disproportionate influence of patients with extreme weights (ie, patients with propensity scores very close to 0 for the exposed and very close to 1 for the unexposed), weights were truncated at the 95th percentile.^19^ Balance of variables between groups was assessed using unweighted and IPTW standardized mean differences (SMDs) using the R package cobalt. An absolute SMD <0.2 was considered indicative of an appreciable reduction in imbalance (Figure 2). Weighted cause-specific Cox regressions were then performed on each imputed data set, and estimates were pooled according to Rubin’s rules; results are presented as HR with 95% CI. Robust standard errors were used to account for the weighting. The proportional hazards assumption was verified using the Schoenfeld residuals. A prespecified stratified analysis according to RoPE score (≥7 vs <7) was performed, with this cutoff based on prior literature;^15^ interaction between treatment effect and RoPE subgroup was tested. A sensitivity analysis excluding patients with missing covariate data (complete case analysis) was conducted to assess robustness. A 2-tailed alpha level of 0.05 was used to define statistical significance. In accordance with established methodology, a *P* value <0.10 was used for interaction tests given their reduced statistical power. Analyses were performed using R version 4.3.2 (R Core Team, 2023).^20^

**Table 1:** Variables Included in the Propensity Score^a^

Sex	0 (0%)
PFO closure indication	0 (0%)
RoPE score^b^	2 (0.2%)
Atrial septal aneurysm	61 (6.5%)
Baseline shunt grade	41 (4.4%)
Device size	67 (7.1%)
Device type	39 (4.1%)
Intraprocedural echo imaging	11 (1.2%)

Values are n (%).

PFO = patent foramen ovale; RoPE = risk of paradoxical embolism.

a A total of 107 patients (11.4%) had at least 1 missing covariate and were handled using multiple imputation.

b The RoPE score (range: 0-10) integrates age, the absence of vascular risk factors (hypertension, diabetes mellitus, current smoking, prior stroke, or transient ischemic attack), and the presence of a cortical infarct on neuroimaging.

## Results

### Baseline and procedural characteristics

As shown in Figure 1, among the 1,245 patients enrolled in the PROLONG registry, 6 were excluded because of device malpositioning requiring either implantation of a second device in situ or surgical intervention, 17 for significant residual shunt, 95 for a baseline indication for long-term antithrombotic therapy unrelated to PFO closure (29 coronary or peripheral artery disease, 48 history of deep vein thrombosis and/or pulmonary embolism, 18 high-risk thrombophilia), and 198 due to missing data on postprocedural APT. The final study population included 940 patients.

Following PFO device closure, 849 patients (90.3%) were discharged on DAPT, 89 (9.5%) on SAPT, and 2 patients (0.2%) without any antithrombotic therapy (one due to recent ischemic stroke with hemorrhagic transformation and the other due to severe thrombocytopenia). DAPT, consisting of aspirin plus a P2Y_12_ inhibitor (ticlopidine until the early 2000s, then clopidogrel as it became widely adopted in Italy), had a mean duration of 4.1 ± 2.6 months. In nearly all cases (98.8%), patients continued with SAPT following DAPT discontinuation, predominantly with aspirin (97.2%). Overall, an APT discontinuation strategy was adopted in 461 patients (49.0%; mean duration 6.5 ± 1.6 months), whereas an APT continuation strategy was used in 479 patients (51.0%; mean duration 12.4 ± 3.5 years).

Baseline and procedural characteristics of the study population, stratified by APT duration, are summarized in Table 2. An APT discontinuation strategy, as opposed to an APT continuation strategy, was more frequently adopted in younger patients (45 ± 12 vs 49 ± 12 years; *P* < 0.001), in women (59.9% vs 49.7%; *P* = 0.002), and in those with a lower prevalence of cardiovascular risk factors, including hypertension (8.0% vs 31.3%; *P* < 0.001), diabetes mellitus (1.5% vs 5.0%; *P* = 0.005), smoking (8.0% vs 16.9%; *P* < 0.001), and hyperlipidemia (9.1% vs 25.7%; *P* < 0.001). Consequently, patients managed with an APT discontinuation strategy had significantly higher RoPE scores (6.5 ± 1.5 vs 6.1 ± 1.8; *P* < 0.001). Moreover, when the index event was ischemic stroke, as opposed to TIA, SE, or silent lesion on magnetic resonance imaging, an APT continuation strategy was more frequently adopted (54.9% vs 7.4%; *P* < 0.001). Regarding procedural characteristics, an APT discontinuation strategy was more common when the procedure was echo-guided (either transesophageal or intracardiac) (98.9% vs 79.8%; *P* < 0.001), when a smaller device was used (23.1 ± 4.3 vs 25.1 ± 4.3 mm; *P* < 0.001), and when an Amplatzer Occluder (Abbott, USA) was implanted (84.8% vs 72.2%; *P* < 0.001) (PFO device details in Supplemental Table 1).

**Table 2:** Baseline and Procedural Characteristics of the Study Population Stratified by Duration of APT After PFO Closure: APT Discontinuation Group (≤12 Months) vs APT Continuation Group (>12 Months)

Variable	Patients With Available Data	Overall (N = 940)	APT Discontinuation (n = 461)	APT Continuation (n = 479)	*P* Value
Age, y	940	47 ± 12	45 ± 12	49 ± 12	**<0.001**
Female	940	514 (54.7%)	276 (59.9%)	238 (49.7%)	**0.002**
Body mass index, kg/m^2^	720	24.7 ± 4.1	24.1 ± 3.8	25.5 ± 4.2	**<0.001**
Hypertension	940	187 (19.9%)	37 (8.0%)	150 (31.3%)	**<0.001**
Diabetes mellitus	940	31 (3.3%)	7 (1.5%)	24 (5.0%)	**0.005**
Active smoking	940	118 (12.6%)	37 (8.0%)	81 (16.9%)	**<0.001**
Hyperlipidemia	939	165 (17.6%)	42 (9.1%)	123 (25.7%)	**<0.001**
History of DVT or PE	828	33 (4.0%)	15 (3.3%)	18 (4.8%)	0.35
Thrombophilia	693	134 (19.3%)	79 (19.4%)	55 (19.3%)	1.00
Migraine	940	280 (29.8%)	136 (29.5%)	144 (30.1%)	0.91
Closure indication: ischemic stroke	940	297 (31.6%)	34 (7.4%)	263 (54.9%)	**<0.001**
RoPE score	938	6.3 ± 1.7	6.5 ± 1.5	6.1 ± 1.8	**<0.001**
Atrial septal aneurysm	879	271 (30.8%)	114 (25.7%)	157 (36.1%)	**<0.001**
Baseline severe shunt	899	514 (57.2%)	252 (56.0%)	262 (59.0%)	0.36
PASCAL classification: probable	925	301 (32.5%)	156 (34.1%)	145 (31.0%)	0.34
Intraprocedural echo imaging	929	824 (88.7%)	455 (98.9%)	369 (79.8%)	**<0.001**
Device size, mm	873	24.1 ± 4.4	23.1 ± 4.3	25.1 ± 4.3	**<0.001**
Device type: Amplatzer	901	707 (78.5%)	379 (84.8%)	328 (72.2%)	**<0.001**
DAPT duration, mo	802	4.1 ± 2.6	3.0 ± 0.6	5.4 ± 3.3	**<0.001**
APT duration, y	940	6.6 ± 6.5	0.5 ± 0.2	12.4 ± 3.5	**<0.001**

Values are mean ± SD or n (%).

APT = antiplatelet therapy; DAPT = dual antiplatelet therapy; DVT = deep vein thrombosis; PASCAL = PFO-associated stroke causal likelihood; PE = pulmonary embolism; other abbreviations as in Table 1.

### Unadjusted survival analysis

**Figure 3:**
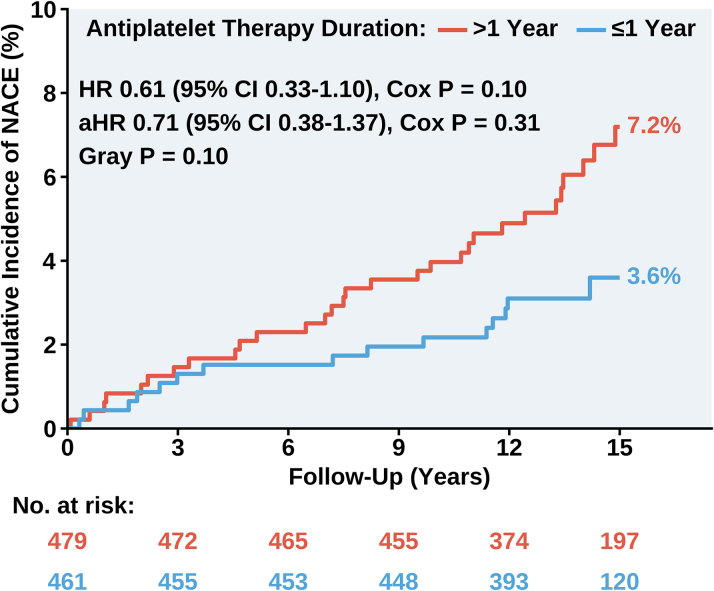
**Cumulative Incidence of Events During Follow-Up According to Antiplatelet Therapy Duration After Patent Foramen Ovale Closure in the Overall Population** aHR = adjusted hazard ratio; NACE = net adverse clinical events.

Follow-up outcomes according to APT duration after PFO closure are shown in Table 3 and Figure 3. The mean follow-up was 14.0 ± 3.1 years (range: 4.2-24.7 years). During follow-up, 47 of 940 patients (5.6%; 0.36 events per 100 patient-years) experienced a NACE: 17 of 461 (3.6%; 0.27 events per 100 patient-years) managed with an APT discontinuation strategy and 30 of 479 (7.2%; 0.44 events per 100 patient-years) managed with an APT continuation strategy (HR: 0.61; 95% CI: 0.33-1.10; Cox *P* = 0.10, Gray *P* = 0.10). Recurrent ischemic events (ischemic stroke, TIA, or SE) occurred in 13 patients (3.1%; 0.21 events per 100 patient-years) managed with an APT discontinuation strategy and in 18 patients (4.3%; 0.26 events per 100 patient-years) managed with an APT continuation strategy, with no significant difference (HR: 0.76; 95% CI: 0.37-1.55; Cox *P* = 0.45, Gray *P* = 0.46). Major bleeding events (BARC ≥3) occurred in 4 patients (0.9%; 0.06 events per 100 patient-years) managed with an APT discontinuation strategy and in 12 patients (2.8%; 0.18 events per 100 patient-years) managed with an APT continuation strategy, with the difference reaching statistical significance (HR: 0.32; 95% CI: 0.10-0.95; Cox *P* = 0.046, Gray *P* = 0.048). A full description of recurrent ischemic events was provided in our previous report, whereas details of major bleeding events are presented in Supplemental Table 2.^16^ Cumulative incidence curves for ischemic events and major bleeding are shown in Supplemental Figure 3.

**Table 3:** Clinical Outcomes According to Antiplatelet Therapy Duration Stratified by RoPE Score: Unweighted and IPTW Analysis

	Overall Population (N = 940)								
	APT Discontinuation (n = 461; 49.0%)		APT Continuation (n = 479; 51.0%)		Unweighted			IPTW	
	Events (Rate)	CIF %	Events (Rate)	CIF %	HR (95% CI)	Cox *P* Value	Gray *P* Value	aHR (95% CI)	Cox *P* Value
NACE	17 (0.27)	3.6	30 (0.44)	7.2	0.61 (0.33-1.10)	0.10	0.10	0.71 (0.38-1.37)	0.31
Ischemic	13 (0.21)	3.1	18 (0.26)	4.3	0.76 (0.37-1.55)	0.45	0.46	1.19 (0.55-2.59)	0.66
Bleeding	4 (0.06)	0.9	12 (0.18)	2.8	0.32 (0.10-0.95)	**0.046**	**0.048**	0.24 (0.07-0.75)	**0.014**

	RoPE score ≥7 (N = 467; 49.7%)								
	APT Discontinuation (n = 251; 54.0%)		APT Continuation (n = 216; 46.0%)		Unweighted			IPTW	
	Events (Rate)	CIF %	Events (Rate)	CIF %	HR (95% CI)	Cox *P* Value	Gray *P* Value	aHR (95% CI)	Cox *P* Value
NACE	5 (0.14)	2.0	12 (0.38)	6.2	0.35 (0.12-0.92)	**0.043**	**0.045**	0.32 (0.11-0.90)	**0.039**
Ischemic	4 (0.12)	1.6	6 (0.19)	3.1	0.58 (0.16-2.04)	0.39	0.39	0.77 (0.22-2.72)	0.68
Bleeding	1 (0.03)	0.4	6 (0.19)	2.8	0.13 (0.02-1.09)	0.057	0.052	0.11 (0.01-0.92)	**0.042**

	RoPE score <7 (N = 473; 50.3%)								
	APT Discontinuation (n = 210; 44.0%)		APT Continuation (n = 263; 56.0%)		Unweighted			IPTW	
	Events (Rate)	CIF %	Events (Rate)	CIF %	HR (95% CI)	Cox *P* Value	Gray *P* Value	aHR (95% CI)	Cox *P* Value
NACE	12 (0.42)	5.5	18 (0.49)	8.1	0.86 (0.41-1.79)	0.68	0.70	1.07 (0.49-2.35)	0.89
Ischemic	9 (0.31)	5.0	12 (0.32)	5.3	0.95 (0.40-2.25)	0.90	0.92	1.49 (0.58-3.78)	0.40
Bleeding	3 (0.10)	1.5	6 (0.16)	2.8	0.63 (0.16-2.53)	0.52	0.52	0.41 (0.10-1.68)	0.22
*P* value for interaction^a^					NACE = 0.19; ischemic = 0.55; bleeding = 0.25			NACE = 0.089; ischemic = 0.42; bleeding = 0.31	

aHR = adjusted HR; BARC = Bleeding Academic Research Consortium; IPTW = inverse probability of treatment weighting; TIA = transient ischemic attack; other abbreviations as in Tables 1 and 2.

**Bold** values indicate statistical significance (*P* < 0.05 for all comparisons except interaction tests, for which *P* < 0.10 was applied).

a *P* for interaction tests the differential treatment effect across RoPE strata (≥7 vs <7). Events (rate) = number of events and event rate per 100 patient-years. CIF % = 15-year cumulative incidence function estimate accounting for the competing risk of death. NACE = net adverse clinical events, defined as a composite of ischemic events (ischemic stroke, TIA, or systemic embolism) and major bleeding (BARC ≥3).

As shown in Table 3 and the Central Illustration, when stratified by RoPE score, patients with RoPE ≥7 appeared to have better outcomes with the APT discontinuation strategy. In this subgroup (n = 467; 49.7% of the population), the APT discontinuation strategy was associated with a significantly lower incidence of NACE compared with the APT continuation strategy (2.0% vs 6.2%; 0.14 vs 0.38 events per 100 patient-years; HR: 0.35; 95% CI: 0.12-0.92; Cox *P* = 0.043, Gray *P* = 0.045), mainly attributable to a lower rate of major bleeding events (0.4% vs 2.8%; 0.03 vs 0.19 events per 100 patient-years; HR: 0.13; 95% CI: 0.02-1.09; Cox *P* = 0.057, Gray *P* = 0.052). Conversely, in patients with RoPE <7 (n = 473; 50.3% of the population), NACE rates were comparable between strategies (5.5% vs 8.1%; 0.42 vs 0.49 events per 100 patient-years; HR: 0.86; 95% CI: 0.41-1.79; Cox *P* = 0.68, Gray *P* = 0.70), with similar rates of both ischemic and bleeding events. The interaction between treatment effect and RoPE score subgroup was tested but did not reach statistical significance (*P* for interaction = 0.19).

**Central Illustration:**
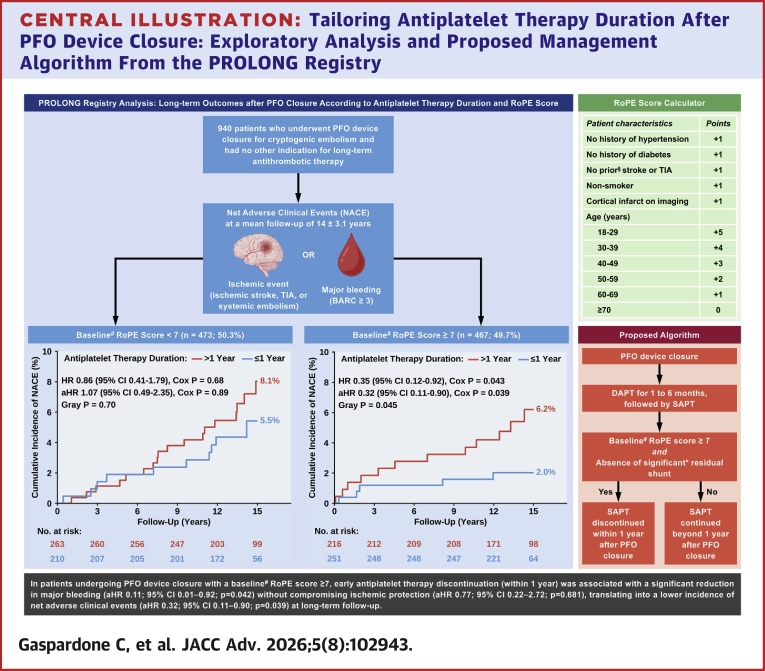
Tailoring Antiplatelet Therapy Duration After PFO Device Closure: Exploratory Analysis and Proposed Management Algorithm From the PROLONG Registry ^§^indicates that the index event prompting patent foramen ovale closure was the patient’s first-ever ischemic stroke or transient ischemic attack. ^#^at the time of patent foramen ovale closure procedure. ∗moderate or severe (grade 2-3) shunt. BARC = Bleeding Academic Research Consortium; SAPT = single antiplatelet therapy with aspirin 75 to 325 mg/d or clopidogrel 75 mg/d; DAPT = dual antiplatelet therapy with aspirin 75 to 325 mg/d plus clopidogrel 75 mg/d; TIA = transient ischemic attack; other abbreviations as in Figures 1 to 3.

### Adjusted survival analysis

A propensity score was created using 8 clinical, echocardiographic, and procedural variables (Table 1). Missing covariate data were observed for device size (7.1%), atrial septal aneurysm (6.5%), baseline shunt grade (4.4%), device type (4.1%), intraprocedural echo imaging (1.2%), and RoPE score (0.2%); no missing data were present for sex and PFO closure indication. Missing data were handled using multiple imputation. The median propensity score values were 0.76 (Q1-Q3: 0.64-0.84) in the APT discontinuation group and 0.25 (Q1-Q3: 0.17-0.56) in the APT continuation group. As shown in Figure 2, baseline SMDs showed imbalances in stroke as closure indication (0.48), device size (0.44), and RoPE score (0.24). After IPTW, all variables showed an appreciable reduction in imbalance, with absolute SMD values below 0.20.

HRs for clinical outcomes were estimated using IPTW Cox proportional hazards regressions. In the overall population, the IPTW analysis confirmed no significant difference in NACE (adjusted HR [aHR]: 0.71; 95% CI: 0.38-1.37; *P* = 0.31) or ischemic events (aHR: 1.19; 95% CI: 0.55-2.59; *P* = 0.66) between the APT discontinuation and continuation strategies. The lower incidence of major bleeding in the APT discontinuation group remained significant after adjustment (aHR: 0.24; 95% CI: 0.07-0.75; *P* = 0.014) (Figure 3, Table 3).

As shown in Table 3 and the Central Illustration, when stratified by RoPE score, the lower incidence of NACE with the APT discontinuation strategy in patients with RoPE ≥7 was confirmed and strengthened after IPTW adjustment. In this subgroup, the APT discontinuation strategy was associated with a significantly lower incidence of NACE (aHR: 0.32; 95% CI: 0.11-0.90; *P* = 0.039), with the difference in major bleeding accounting for most of this reduction (aHR: 0.11; 95% CI: 0.01-0.92; *P* = 0.042). Conversely, in patients with RoPE <7, no difference was observed for any outcome. The interaction between treatment effect and RoPE subgroup reached statistical significance for NACE (*P* for interaction = 0.089).

A sensitivity analysis without imputation, thus restricted to patients with complete covariate data (n = 833, 89% of the cohort), confirmed these findings, with the lower NACE incidence with the APT discontinuation strategy in patients with RoPE ≥7 remaining significant (aHR: 0.17; 95% CI: 0.05-0.63; *P* = 0.006 for NACE) (Supplemental Figure 1, Supplemental Table 3).

## Discussion

In this large multicenter registry with extended follow-up, an APT discontinuation strategy (≤12 months) after PFO device closure was associated with a significant reduction in major bleeding without a concomitant increase in ischemic events. This reduction in NACE was confined to patients with a RoPE score ≥7, whereas no significant difference was observed in those with lower scores.

### APT after PFO closure: current guidelines and evidence gaps

Current guidelines do not provide definitive recommendations regarding the optimal duration of APT after PFO device closure.^1^, ^2^, ^3^ This uncertainty stems from the lack of dedicated randomized controlled trials (RCTs) specifically addressing this issue. Existing guidance is largely extrapolated from the trial protocols of pivotal studies—CLOSE, REDUCE, RESPECT, and DEFENSE-PFO—that compared device closure with medical therapy.^9^ Most expert consensus statements recommend a short course of DAPT lasting 1 to 6 months, followed by long-term SAPT.^8^^,^^9^ In the absence of robust evidence-based guidance, clinical practice remains highly variable. This heterogeneity was also reflected in the PROLONG registry, where the use of APT discontinuation and continuation strategies were almost equally adopted (49% vs 51%), underscoring the lack of a clear clinical consensus. Although current expert guidance generally favors prolonged SAPT after the DAPT phase, some observational studies have reported favorable outcomes even after discontinuation of APT within 6 to 12 months postimplantation.^10^, ^11^, ^12^

### Toward a tailored approach: rationale for early APT discontinuation after PFO closure

APT after PFO device closure serves a dual purpose^1^: reducing the risk of device-related thrombosis and^2^ providing secondary prevention of ischemic events unrelated to the PFO itself.

The primary rationale for using APT for PFO device implantation is to prevent device-related thrombosis. Although studies have reported conflicting findings, most evidence agrees that device endothelial coverage begins as early as 1 month after implantation and is typically complete within 3 to 6 months.^4^, ^5^, ^6^ Consistently, most reported thrombotic events occur within the first few months after the procedure, while events beyond the first year are extremely rare.^7^ In the PROLONG registry, device-related thrombosis occurred in only 3 patients (0.3%), all within 6 months after implantation (at 1.6, 3.7, and 4.3 months), with one patient on DAPT and 2 on SAPT. One patient experienced an ischemic stroke associated with a thrombus on the left atrial disc, requiring hospitalization and intravenous anticoagulation. In the remaining 2 cases, thrombi were incidentally detected by echocardiography in asymptomatic patients (one on the left disc and one on the right disc) and were successfully managed with outpatient oral anticoagulation. Complete thrombus resolution was documented in all 3 patients at 1-month follow-up. These findings reinforce the concept that the risk of device-related thrombosis is low and largely confined to the early postimplantation period, supporting the rationale for a limited course of APT.

The second rationale for extending APT after PFO closure relates to the prevention of recurrent ischemic events unrelated to the PFO itself. Because paradoxical embolism is rarely documented with direct evidence (ie, thrombus in transit), the causal relationship between a PFO and an embolic event remains probabilistic. The RoPE score is a validated tool that estimates the probability that a PFO identified in the setting of an otherwise cryptogenic embolism is causally related to the event rather than an incidental finding.^14^ A higher RoPE score corresponds to a greater likelihood of a PFO-related embolism and is typically observed in younger patients, in the absence of traditional cardiovascular risk factors, with neuroimaging showing a cortical infarct (see Central Illustration for RoPE score components). In patients with a RoPE score ≥7, the probability that the index event was PFO-related exceeds 70% to 80%. In these individuals, PFO closure is thought to eliminate the embolic source, and the rationale for extending APT therapy becomes less compelling. Conversely, in patients with lower RoPE scores—such as 5 or 6, corresponding to a probability of approximately 50%—the embolic event may have been unrelated to the PFO, and latent mechanisms (eg, atrial fibrillation, atherosclerosis) cannot be excluded. In such cases, prolonged APT as a secondary preventive strategy may be justified.^14^^,^^15^ Supporting this rationale, patients with a RoPE score <7 in the PROLONG registry had more than double the risk of recurrent ischemic events compared with those with a score ≥7 (0.32 vs 0.15 events per 100 patient-years; HR: 2.1; *P* = 0.042).

The third key consideration of this discussion is the bleeding risk inherently associated with APT. In our registry, 16 patients (1.7%; 0.12 events per 100 patient-years) experienced a major bleeding event (BARC ≥3) during follow-up, including 2 fatal hemorrhages (see Supplemental Table 2 for details). Notably, 12 of 16 bleeding events (75%) occurred in patients managed with an APT continuation strategy, translating to a threefold higher bleeding risk compared with the APT discontinuation strategy (0.18 vs 0.06 events per 100 patient-years; HR: 3.1; *P* = 0.046). In patients with a RoPE score ≥7, the lower incidence of NACE with the APT discontinuation strategy was primarily attributable to a lower incidence of bleeding, suggesting that in this low-ischemic-risk population, prolonged APT may confer more harm than benefit (NACE: 0.14 vs 0.38 events per 100 patient-years; aHR: 0.32; 95% CI: 0.11-0.90; *P* = 0.039). In line with our findings, a large prospective registry reported a major bleeding rate of 0.16 events per 100 patient-years, with all cases occurring in patients on APT.^11^ Notably, our bleeding rate is likely underestimated due to the retrospective design, as acknowledged in the Limitations. Indeed, a meta-analysis of 6 major PFO RCTs reported a substantially higher rate (0.46 per 100 patient-years), further underscoring that even in this young population, the bleeding burden associated with prolonged APT is clinically relevant.^13^

In conclusion, A) the rarity of device-related thrombosis beyond the first year; B) the limited need for secondary prevention in patients with PFO-related events (RoPE score ≥7); and C) the bleeding risk associated with prolonged APT, provide a strong pathophysiological rationale for our findings. In fact, among patients with a baseline RoPE score ≥7 and effective PFO device closure, the bleeding risk associated with extended APT may ultimately outweigh the risk of recurrent ischemic events. Therefore, although the present findings are exploratory and hypothesis-generating given the observational design and limited statistical power, they provide a pathophysiological and clinical rationale for a risk-stratified APT strategy after PFO closure, as illustrated in the Central Illustration. This framework is not intended as a practice-changing recommendation, but rather as a hypothesis to be tested in dedicated randomized trials.

### Study limitations

This analysis of the PROLONG registry shares the limitations of the original registry, which have been previously described in detail.^16^ These limitations, inherent to the observational retrospective nature of the study, include missing baseline data, loss to follow-up, the absence of a standardized follow-up protocol, and the lack of a dedicated core laboratory for outcome adjudication. Furthermore, the enrollment period (1999-2013), while conferring the advantage of an extended follow-up of nearly 15 years, may limit the generalizability of our findings to contemporary PFO closure practice. Specific to the present analysis, some additional limitations warrant consideration. First, from the original 1,245 patients, 198 (15.9%) were excluded due to missing data on postprocedural APT (Figure 1), which may have introduced a selection bias. Second, the duration and type of APT were left to the discretion of the treating physician and center, reflecting real-world practice but potentially introducing a treatment bias. Although IPTW was applied to account for measured confounders, the lack of randomization does not allow to completely exclude the effect of unmeasured confounding factors. Nevertheless, the consistency of results across multiple analytical approaches (unweighted, IPTW, and sensitivity analysis without imputation) supports the robustness of our findings. Furthermore, although IPTW achieved an appreciable reduction in baseline imbalances, some covariates, most notably ischemic stroke as the indication for PFO closure, retained an SMD above the more stringent threshold of 0.1, which should be acknowledged as a residual source of potential confounding. Third, patients managed with an APT continuation strategy also tended to receive a longer duration of DAPT compared with those managed with an APT discontinuation strategy (5.4 vs 3.0 months; *P* < 0.001). However, DAPT duration was not associated with NACE in Cox regression analysis (HR 1.05 per month increase, 95% CI: 0.93-1.17; *P* = 0.42), suggesting that variations in the initial DAPT period had no meaningful impact on long-term outcomes (Supplemental Table 4). This finding is further supported by the landmark analysis (Supplemental Table 5, Supplemental Figure 2), which excluded the early DAPT phase and yielded results consistent with the main analysis. Fourth, bleeding risk factors (eg, HAS-BLED score) were not systematically assessed, limiting the ability to account for baseline bleeding risk in the comparative analyses. Fifth, nearly all patients (97.2%) continued aspirin after DAPT, precluding any meaningful comparison between aspirin and clopidogrel. Similarly, subgroup analyses based on device type were not feasible, as Amplatzer Occluders were used in nearly 80% of cases. Sixth, the binary classification of APT duration based on a 12-month cutoff (median APT duration of the cohort) is inherently arbitrary in the absence of dedicated randomized trials and may not fully capture the heterogeneity of antiplatelet management in clinical practice. Finally, although 940 patients were followed for 14 years, the overall event rate was fortunately low (47 NACE events, 5.6%), rendering the study underpowered to draw robust conclusions and making the results statistically fragile, particularly in subgroup analyses. Our findings should therefore be considered exploratory and hypothesis-generating rather than practice-changing and warrant confirmation in dedicated randomized studies.

## Conclusions

In patients with a RoPE score ≥7, early discontinuation of APT (within 12 months) after effective PFO closure was associated with a lower incidence of NACE at long-term follow-up. These findings underscore the importance of tailoring APT duration to individual risk profiles. Nevertheless, given the observational nature and limited statistical power of this analysis, these results should be considered hypothesis-generating, and dedicated randomized studies are warranted to confirm these findings.

Perspectives **COMPETENCY IN MEDICAL KNOWLEDGE:** In patients with a RoPE score ≥7, early discontinuation of APT after effective PFO closure may reduce major bleeding without compromising ischemic protection, supporting a risk-stratified rather than uniform long-term APT strategy.

**TRANSLATIONAL OUTLOOK:** Dedicated RCTs stratified by risk profile, including RoPE score, are needed to validate early APT discontinuation as a safe and effective strategy and to support future guideline recommendations.

## Funding support and author disclosures

The authors have reported that they have no relationships relevant to the contents of this paper to disclose.
