# Supplementary material for: Tailoring Antiplatelet Therapy Duration After PFO Closure: Insights From the PROLONG Registry
Source: JACC Adv. 2026 Jun 30;5(8):102943. doi: 10.1016/j.jacadv.2026.102943 (PMC13343141; doi:10.1016/j.jacadv.2026.102943)

Supplementary Table 1. Patent Foramen Ovale Device Type (n = 901)

**PFO Device Type (n = 901)**	**n (%)**
Amplatzer (AGA-St. Jude-Abbott)	707 (78.5)
Figulla Flex II (Occlutech)	41 (4.6)
CardioSEAL (NMT Medical)	33 (3.7)
INTRASEPT (Cardia)	30 (3.3)
Premere (St. Jude-Abbott)	29 (3.2)
BioSTAR (NMT Medical)	18 (2.0)
CARDIOFORM Septal Occluder (W.L. Gore)	26 (2.9)
FlatStent (Coherex Medical)	8 (0.9)
Nit-Occlud PFO (PFM Medical)	7 (0.8)
Other device	1 (0.1)
Values are n (%).	

Supplementary Table 2. Detailed Information of Major Bleeding Events (BARC ≥ 3) after PFO Closure

#	PFO closure indication	Baseline RoPE score	Baseline RoPE score ≥ 7	PFO closure device	Bleeding event type (BARC)	Bleeding event details	Time between PFO closure and event (years)	APT group	Antithrombotic therapy at the event time
1	TIA	5	No	Amplatzer	3A	Lower GI bleeding	7	Short	None
2	TIA	5	No	Amplatzer	3A	Upper GI bleeding	2	Short	None
3	TIA	4	No	Amplatzer	5A	Fatal hemorrhagic shock	11	Short	None
4	Silent lesions at MRI	9	Yes	Amplatzer	3A	Unspecified	12	Short	None
5	Stroke	5	No	Cardioseal	3B	Upper GI bleeding	14	Long	ASA
6	Stroke	7	Yes	Amplatzer	3A	Hemarthrosis	5	Long	ASA
7	Stroke	4	No	Amplatzer	3A	Hematuria	3	Long	ASA
8	TIA	4	No	Intrasept (cardia)	5B	Fatal intracranial hemorrhage	12	Long	ASA
9	TIA	9	Yes	Amplatzer	3B	Retroperitoneal	1	Long	ASA + Clopidogrel
10	TIA	4	No	Amplatzer	3B	Severe anemia – oncologic patient	10	Long	ASA
11	Stroke	7	Yes	Amplatzer	3A	Upper GI bleeding	3	Long	ASA
12	TIA	8	Yes	Amplatzer	3A	Hemoptysis	14	Long	ASA
13	TIA	7	Yes	Amplatzer	3A	Unspecified	7	Long	None
14	TIA	2	No	Unknown	3C	Intracranial hemorrhage	8	Long	ASA
15	TIA	8	Yes	Amplatzer	3A	Lower GI bleeding	2	Long	ASA
16	TIA	6	No	Amplatzer	3A	Upper GI bleeding	13	Long	ASA

BARC = Bleeding Academic Research Consortium; PFO = patent foramen ovale; TIA = transient ischemic attack; MRI = magnetic resonance imaging; GI = gastrointestinal; APT = antiplatelet therapy; ASA = acetylsalicylic acid.

SENSITIVITY ANALYSIS WITHOUT IMPUTATION (N = 833, 89% OF THE COHORT)

Supplementary Figure 1. Covariate Balance Before and After IPTW in the Complete-Case Sensitivity Cohort (n = 833)

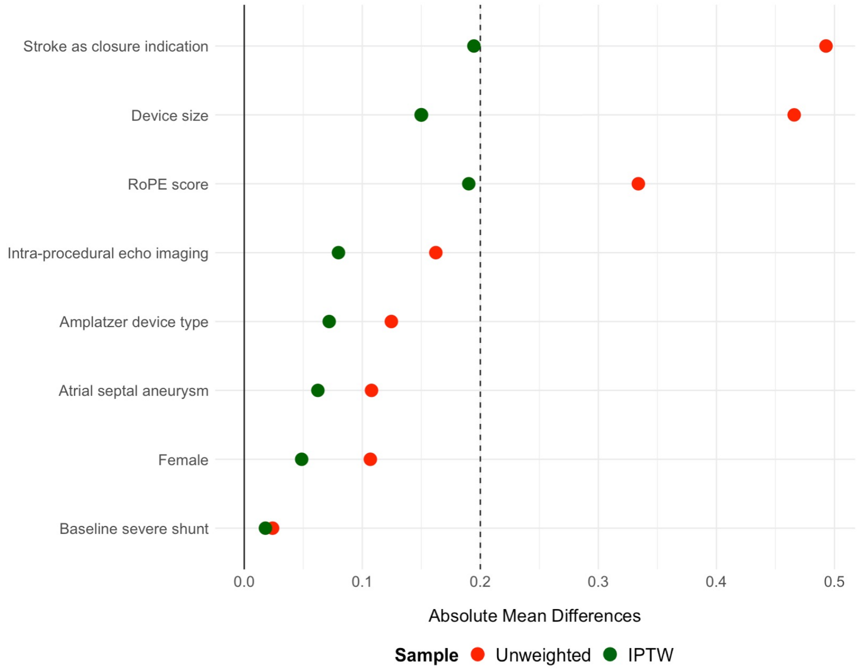

Red dots indicate standardized mean differences (SMDs) at baseline; green dots indicate SMDs after weighting. The dashed line indicates the threshold of 0.2; values below this threshold are indicative of an appreciable reduction in imbalance between groups.

Supplementary Table 3. Clinical Outcomes According to Antiplatelet Therapy Duration Stratified by RoPE Score in the Complete-Case Sensitivity Cohort (n = 833): Unweighted and IPTW Analysis

**Overall Population (n = 833)**									
	**APT Discontinuation (n = 442; 53.0%)**		**APT Continuation (n = 391; 47.0%)**		**Unweighted**			**IPTW**	
	**Events (Rate)**	**CIF %**	**Events (Rate)**	**CIF %**	**HR (95% CI)**	**Cox P**	**Gray P**	**aHR (95% CI)**	**Cox P**
**NACE**	14 (0.23)	3.1	28 (0.50)	8.5	0.45 (0.24–0.86)	**0.015**	**0.014**	0.56 (0.27–1.14)	0.11
**Ischemic**	10 (0.16)	2.6	17 (0.30)	5.1	0.52 (0.24–1.13)	0.099	0.098	0.90 (0.38–2.12)	0.81
**Bleeding**	4 (0.07)	0.9	11 (0.19)	3.2	0.32 (0.10–1.01)	0.051	**0.041**	0.21 (0.07–0.70)	**0.010**
**RoPE Score ≥ 7 (n = 402; 48.0%)**									
	**APT Discontinuation (n = 241; 60.0%)**		**APT Continuation (n = 161; 40.0%)**		**Unweighted**			**IPTW**	
	**Events (Rate)**	**CIF %**	**Events (Rate)**	**CIF %**	**HR (95% CI)**	**Cox P**	**Gray P**	**aHR (95% CI)**	**Cox P**
**NACE**	3 (0.09)	1.3	11 (0.47)	7.9	0.18 (0.05–0.64)	**0.008**	**0.003**	0.17 (0.05–0.63)	**0.006**
**Ischemic**	2 (0.06)	0.8	5 (0.21)	3.6	0.27 (0.05–1.37)	0.11	0.089	0.40 (0.08–2.08)	0.28
**Bleeding**	1 (0.03)	0.4	6 (0.25)	3.7	0.11 (0.01–0.91)	**0.041**	**0.013**	0.08 (0.01–0.73)	**0.025**
**RoPE Score < 7 (n = 431; 52.0%)**									
	**APT Discontinuation (n = 201; 47.0%)**		**APT Continuation (n = 230; 53.0%)**		**Unweighted**			**IPTW**	
	**Events (Rate)**	**CIF %**	**Events (Rate)**	**CIF %**	**HR (95% CI)**	**Cox P**	**Gray P**	**aHR (95% CI)**	**Cox P**
**NACE**	11 (0.40)	5.2	17 (0.53)	9.0	0.76 (0.35–1.62)	0.47	0.48	0.94 (0.41–2.17)	0.88
**Ischemic**	8 (0.29)	4.7	12 (0.37)	6.2	0.76 (0.31–1.86)	0.55	0.56	1.21 (0.46–3.20)	0.70
**Bleeding**	3 (0.11)	1.5	5 (0.15)	2.8	0.69 (0.16–2.90)	0.61	0.61	0.44 (0.10–1.92)	0.27
**P for interaction***					NACE = 0.071; Ischemic = 0.28; Bleeding = 0.16			NACE = **0.036**; Ischemic = 0.26; Bleeding = 0.22	

* P for interaction tests the differential treatment effect across RoPE strata (≥7 vs <7). Events (Rate) = number of events and event rate per 100 patient-years. CIF % = 15-year cumulative incidence function estimate accounting for the competing risk of death. APT = antiplatelet therapy; NACE = net adverse clinical events, defined as a composite of ischemic events (ischemic stroke, TIA, or systemic embolism) and major bleeding (BARC ≥3); HR = hazard ratio; aHR = adjusted hazard ratio; CI = confidence interval; IPTW = inverse probability of treatment weighting; RoPE = Risk of Paradoxical Embolism. Bold values indicate statistical significance (P < 0.05 for all comparisons except interaction tests, for which P < 0.10 was applied).

Supplementary Table 4. Univariable predictors of NACE at Follow-Up

	**No. of Patients with Available Data**	**No NACE (n = 893)**	**NACE (n = 47)**	**HR (95% CI)**	**p-value**
Age, years	940	47 ± 12	49 ± 12	1.02 (0.99-1.04)	0.19
Female	940	488 (55%)	26 (55%)	1.03 (0.58-1.83)	0.92
Body mass index, kg/m^2	720	24.6 ± 4.1	25.6 ± 3.9	1.05 (0.97-1.14)	0.24
Hypertension	940	174 (19%)	13 (28%)	1.64 (0.87-3.11)	0.13
Diabetes mellitus	940	30 (3.4%)	1 (2.1%)	0.64 (0.09-4.65)	0.66
Active smoking	940	109 (12%)	9 (19%)	1.64 (0.79-3.40)	0.18
Hyperlipidemia	939	152 (17%)	13 (28%)	1.85 (0.97-3.52)	0.060
History of DVT or PE	828	31 (3.9%)	2 (4.7%)	1.14 (0.28-4.70)	0.86
Thrombophilia	693	126 (19%)	8 (30%)	1.64 (0.72-3.75)	0.24
Migraine	940	264 (30%)	16 (34%)	1.20 (0.66-2.20)	0.55
Closure indication: ischemic stroke	940	279 (31%)	18 (38%)	1.27 (0.71-2.30)	0.42
RoPE score	938	6.3 ± 1.7	5.8 ± 1.7	0.83 (0.71-0.96)	**0.031**
RoPE score ≥ 7	938	450 (51%)	17 (36%)	0.55 (0.29-0.97)	**0.040**
Atrial septal aneurysm	878	256 (31%)	15 (37%)	1.23 (0.65-2.32)	0.53
Baseline severe shunt	894	490 (57%)	24 (65%)	1.38 (0.70-2.71)	0.35
PASCAL classification: probable	925	292 (33%)	9 (20%)	0.52 (0.25-1.09)	0.084
Intra-procedural echo imaging	928	784 (89%)	40 (87%)	0.81 (0.34-1.91)	0.63
Device size, mm	870	24.1 ± 4.4	25.4 ± 3.7	1.06 (0.98-1.14)	0.053
Device type: Amplatzer	900	673 (79%)	34 (79%)	1.00 (0.48-2.10)	0.99
DAPT duration, months	802	4.1 ± 2.5	4.6 ± 3.0	1.05 (0.93-1.17)	0.42
APT short duration	940	444 (50%)	17 (36%)	0.61 (0.33-1.10)	0.10

Values are mean ± SD or n (%). NACE = net adverse clinical events, defined as a composite of ischemic events (ischemic stroke, transient ischemic attack, or systemic embolism) and major bleeding (BARC ≥ 3). DVT = deep vein thrombosis; PE = pulmonary embolism; RoPE = risk of paradoxical embolism; PASCAL = PFO-associated stroke causal likelihood; DAPT = dual antiplatelet therapy; APT = antiplatelet therapy

LANDMARK ANALYSIS STARTING AT DAPT DISCONTINUATION (N = 940, 100% OF THE COHORT)

Supplementary Table 5. Clinical Outcomes According to Antiplatelet Therapy Duration Stratified by RoPE Score, Starting at DAPT Discontinuation (Landmark Analysis, n = 940): Unweighted and IPTW Analysis

**Overall Population (n = 940)**									
	**APT Discontinuation (n = 461; 49.0%)**		**APT Continuation (n = 479; 51.0%)**		**Unweighted**			**IPTW**	
	**Events (Rate)**	**CIF %**	**Events (Rate)**	**CIF %**	**HR (95% CI)**	**Cox P**	**Gray P**	**aHR (95% CI)**	**Cox P**
**NACE**	17 (0.28)	3.6	28 (0.42)	6.8	0.65 (0.36–1.20)	0.17	0.18	0.80 (0.41–1.56)	0.51
**Ischemic**	11 (0.18)	2.7	17 (0.25)	4.1	0.68 (0.32–1.46)	0.32	0.33	1.13 (0.49–2.59)	0.78
**Bleeding**	3 (0.05)	0.7	10 (0.15)	2.4	0.31 (0.09–1.14)	0.078	0.064	0.23 (0.06–0.84)	**0.027**
**RoPE Score ≥ 7 (n = 467; 49.7%)**									
	**APT Discontinuation (n = 251; 54.0%)**		**APT Continuation (n = 216; 46.0%)**		**Unweighted**			**IPTW**	
	**Events (Rate)**	**CIF %**	**Events (Rate)**	**CIF %**	**HR (95% CI)**	**Cox P**	**Gray P**	**aHR (95% CI)**	**Cox P**
**NACE**	5 (0.15)	2.0	10 (0.32)	5.3	0.44 (0.15–1.27)	0.13	0.12	0.43 (0.14–1.31)	0.14
**Ischemic**	3 (0.09)	1.2	5 (0.16)	2.6	0.52 (0.12–2.18)	0.37	0.37	0.70 (0.17–2.94)	0.63
**Bleeding**	0 (0.00)	0.0	4 (0.13)	1.9	NE	NE	**0.030**	NE	NE
**RoPE Score < 7 (n = 473; 50.3%)**									
	**APT Discontinuation (n = 210; 44.0%)**		**APT Continuation (n = 263; 56.0%)**		**Unweighted**			**IPTW**	
	**Events (Rate)**	**CIF %**	**Events (Rate)**	**CIF %**	**HR (95% CI)**	**Cox P**	**Gray P**	**aHR (95% CI)**	**Cox P**
**NACE**	12 (0.43)	5.4	18 (0.50)	8.0	0.86 (0.41–1.78)	0.68	0.70	1.08 (0.49–2.40)	0.85
**Ischemic**	8 (0.29)	4.5	12 (0.34)	5.3	0.84 (0.34–2.06)	0.71	0.72	1.40 (0.53–3.67)	0.50
**Bleeding**	3 (0.11)	1.4	6 (0.16)	2.8	0.63 (0.16–2.52)	0.52	0.52	0.40 (0.10–1.63)	0.20
**P for interaction***					NACE = 0.35; Ischemic = 0.60; Bleeding = NE			NACE = 0.20; Ischemic = 0.44; Bleeding = NE	

* P for interaction tests the differential treatment effect across RoPE strata (≥7 vs <7). The landmark analysis excluded patients with adverse events occurring during the initial dual antiplatelet therapy phase, with time zero set at the end of the DAPT period. Events (Rate) = number of events and event rate per 100 patient-years. CIF % = 15-year cumulative incidence function estimate accounting for the competing risk of death. NE = not estimable (no events in the APT Discontinuation arm preclude a valid Cox estimate). APT = antiplatelet therapy; NACE = net adverse clinical events, defined as a composite of ischemic events (ischemic stroke, TIA, or systemic embolism) and major bleeding (BARC ≥3); HR = hazard ratio; aHR = adjusted hazard ratio; CI = confidence interval; IPTW = inverse probability of treatment weighting; RoPE = Risk of Paradoxical Embolism. Bold values indicate statistical significance (P < 0.05 for all comparisons except interaction tests, for which P < 0.10 was applied).

Supplementary Figure 2. Cumulative Incidence of NACE According to Antiplatelet Therapy Duration, Stratified by RoPE Score, Starting at DAPT Discontinuation (Landmark Analysis, n = 940)

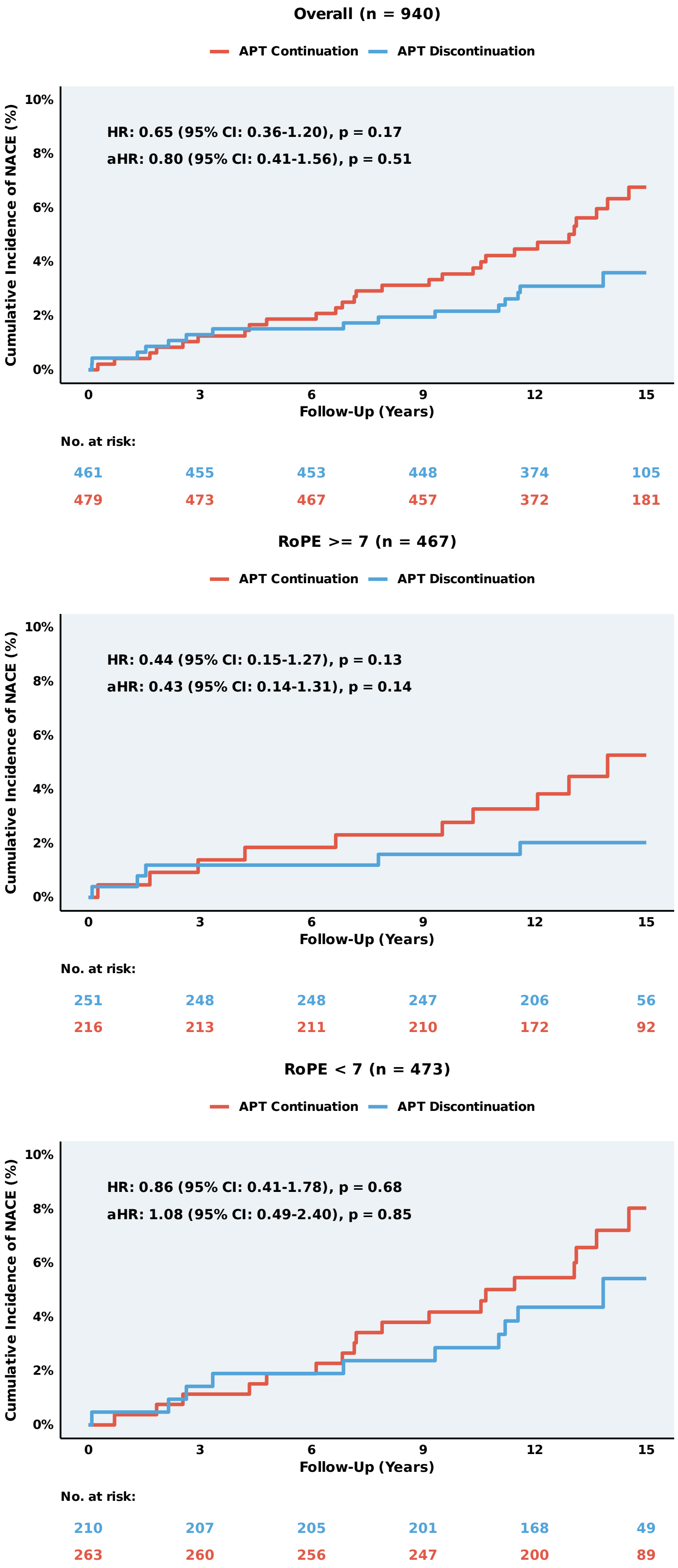

Time zero was set at DAPT discontinuation, excluding events occurring during the initial DAPT phase. NACE = net adverse clinical events; PFO = patent foramen ovale; DAPT = dual antiplatelet therapy.

Supplementary Figure 3. Cumulative Incidence of Ischemic Events and Major Bleeding According to Antiplatelet Therapy Duration, Stratified by RoPE Score (n = 940)

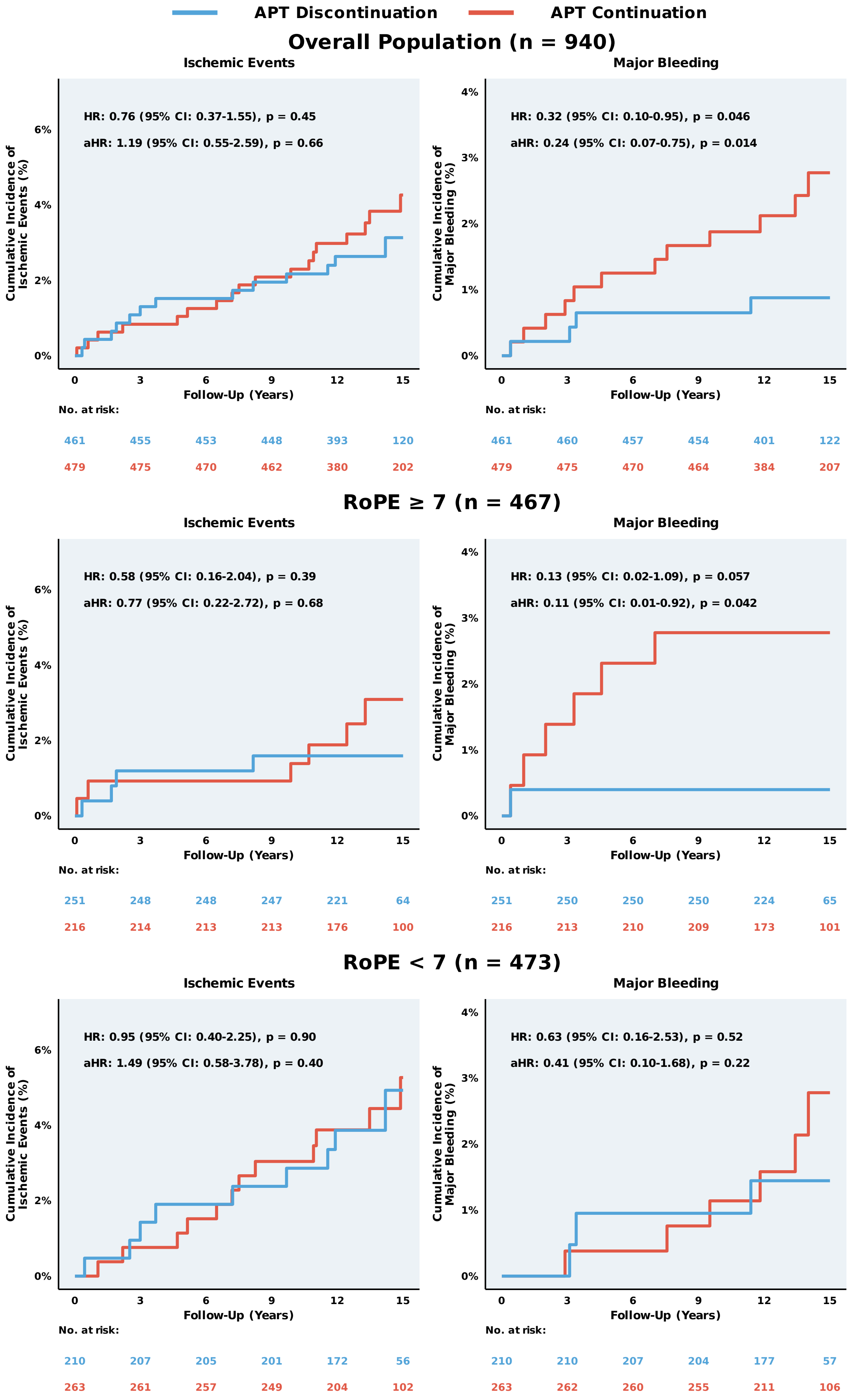

Supplement: Supplemental_Material [file mmc1.pdf]
